# Resilience in symptom response to cancer treatment: a new lens for examining variable symptom trajectories in older adults with advanced cancer

**DOI:** 10.1093/oncolo/oyag094

**Published:** 2026-03-20

**Authors:** Zhihong Zhang, Eva Culakova, Kah Poh Loh, Kathi L Heffner, Mostafa Mohamed, Rachael G Tylock, Megan Wells, Fiona A Stauffer, Supriya Mohile, Marie Flannery

**Affiliations:** University of Rochester, School of Nursing, Rochester, NY, 14642, United States; University of Rochester Medical Center, Department of Surgery, Rochester, NY, 14642, United States; University of Rochester Medical Center, Division of Hematology/Oncology, Department of Medicine and Wilmot Cancer Institute, Rochester, NY, 14642, United States; University of Rochester, School of Nursing, Rochester, NY, 14642, United States; University of Rochester Medical Center, Division of Hematology/Oncology, Department of Medicine and Wilmot Cancer Institute, Rochester, NY, 14642, United States; University of Rochester Medical Center, Division of Hematology/Oncology, Department of Medicine and Wilmot Cancer Institute, Rochester, NY, 14642, United States; University of Rochester Medical Center, Division of Hematology/Oncology, Department of Medicine and Wilmot Cancer Institute, Rochester, NY, 14642, United States; University of Rochester Medical Center, Division of Hematology/Oncology, Department of Medicine and Wilmot Cancer Institute, Rochester, NY, 14642, United States; University of Rochester Medical Center, Division of Hematology/Oncology, Department of Medicine and Wilmot Cancer Institute, Rochester, NY, 14642, United States; University of Rochester, School of Nursing, Rochester, NY, 14642, United States

**Keywords:** resilience, resilience concept model, symptom, cancer treatment, functional status, older adults

## Abstract

**Background:**

Symptom trajectories vary among patients during cancer treatment. The National Institutes of Health resilience model, which defines resilience as the ability to resist, recover, or grow from stressors, may be useful in understanding these variations. This study examined variations in symptom trajectories through the lens of resilience among older adults with advanced cancer.

**Methods:**

This longitudinal quantitative study included patients aged 70 and older with advanced cancer who were receiving treatment regimens associated with a high risk of treatment-related toxicities, using data from the GAP70+ study (NCT02054741). A summary severity score of 24 symptoms from the Patient-Reported Outcomes version of the Common Terminology Criteria for Adverse Events and functional status (measured by instrumental activities of daily living) were assessed prior to the treatment regimen initiation, at 4-6 weeks, 3 months, and 6 months later. Symptom trajectories were estimated using growth mixture models. Resilience was indicated by trajectories of resisting, recovering, or growing (ie, improving from moderate/severe to none/mild severity). Its association with functional status was examined using longitudinal linear mixed models.

**Results:**

The study included 710 patients (average age 77.2, 43.2% female, 88.0% receiving chemotherapy). Overall, 17.7% of patients showed resilience in symptom response, which was associated with higher baseline functional status (13.2 vs 12.0, *P* **<**.001) and stable functioning over 6 months. In contrast, non-resilience in symptom response was associated with a functional decline of 0.7-0.9 points without recovery by 6 months (*P***<**.001).

**Conclusion:**

Resilience in symptom response provides a novel perspective on symptom trajectory variations. Future research should explore underlying contributing factors to inform interventions that promote resilient symptom responses.

Implications for Practice Symptom trajectories during cancer treatment vary widely among older adults. This study introduces a novel application of the NIH resilience model to characterize these trajectories in older adults receiving cancer treatment. Resilience in symptom response was defined by trajectories of resisting, recovering, or improving symptoms during treatment, and was associated with higher functional status and greater stability over time. These findings highlight the potential for leveraging resilience-informed symptom trajectories to guide personalized and supportive interventions in oncology care.

## Introduction

Individuals aged 65 and older, who account for over half of new cancer diagnoses,^1^ often experience increased symptoms during cancer treatment due to both the cancer and its treatment.^2^ Treatment-related symptoms (ie, symptomatic toxicities) are often more severe in those with aging-related conditions and advanced stages of cancer.^3–6^ Research across the broader adult cancer population indicates that while symptom severity tends to increase on average during cancer treatment,^7–13^ responses vary considerably across individuals. Some individuals tolerate cancer treatment well with no/mild symptoms^14–17^; others experience increased symptoms in response to cancer treatment, with some recovering after treatment completion and others suffering from persistent symptoms without recovering.^14–17^ The varying symptom trajectories indicate individual heterogeneity in symptom response to cancer treatment. Thus, understanding these variable symptom trajectories in older adults with cancer is crucial, as this information can inform targeted interventions aimed at managing symptoms more effectively.

The National Institutes of Health Resilience Concept Model (NIH-RCM), proposed by the Trans-National Institutes of Health (Trans-NIH) Resilience Working Group, is an ideal framework for examining variable responses to cancer treatment.^18^^,^^19^ According to the NIH-RCM, when individuals face a challenge or stressor, they may exhibit resilience or non-resilience in their responses. When adapting the NIH-RCM to individuals’ symptom response to the stressor of cancer treatment, resilience in symptom response to cancer treatment is defined as a dynamic process of positive adaptation in the face of cancer treatment that results in none or mild symptom severity (a positive outcome) at its end, reflecting the ability to resist, recover, or grow (improve) from cancer treatment. Individuals with resilience in symptom response to cancer treatment may follow one of three trajectories: “resist” (maintain none/mild symptom severity), “recover” (symptom severity increases from none/mild to moderate/severe, then returns to none/mild), or “grow” (improve from moderate/severe to none/mild symptom severity). In contrast, individuals without resilience may experience moderate to severe long-term symptoms with only partial or no recovery during cancer treatment. Although NIH-RCM is a promising model for explaining the variation in symptom trajectories, it has not been utilized and validated in empirical studies.

Functional decline is common among older adults with cancer, particularly during treatment.^20–24^ Prior research has identified subgroups of patients who follow resilience trajectories (eg, more stable or recovery patterns), which are associated with better outcomes, ^25^^,^^26^ such as higher functional status throughout treatment.^27^^,^^28^ These findings suggest that resilience in symptom response may also be associated with better functional stability over the course of treatment. Thus, the aims of this study were (1) to identify resilience in symptom response to cancer treatment according to the NIH-RCM using data from a national clinical trial among older adults with advanced cancer and (2) to examine the association between resilience in symptom response to cancer treatment and functional status over 6 months.

## Methods

### Study design and sample

This study was a longitudinal quantitative research design using existing data from a national cluster-randomized study (Geriatric Assessment for Patients 70 years and older [GAP 70+], ClinicalTrials.gov, NCT02054741, PI: Mohile). Patients in the GAP 70+ study were adults aged 70 and older with advanced cancer who had at least one aging-related condition other than polypharmacy, as identified through impairments in geriatric assessment, and were scheduled to begin a new cancer treatment regimen associated with a high risk of toxicity.^29^ We included patients enrolled in the GAP 70+ study who completed at least one symptom assessment by the Patient-Reported Outcomes Version of the Common Terminology Criteria for Adverse Events (PRO-CTCAE). This decision was based on preliminary analyses among patients alive at 6 months, in which only 17 patients had a single assessment. Resilience rates were nearly identical between patients with at least one versus at least two assessments (data not shown). Given the minimal impact on resilience classification and the small difference in sample size, including patients with at least one assessment was appropriate and allowed us to maximize the analytic sample. IRB approval was obtained from the University of Rochester and all participating practice clusters, and all participating practice sites received approval from their respective institutional review boards.

### Specification of resilience in symptom response to cancer treatment

This study used the NIH-RCM as a theoretical framework, which describes resilience in the context of three components (stressor, system, and response) and defines it as the system’s ability to resist, recover, or grow in response to a stressor, based on changes from baseline.^25^ In this study, the *stressor* was specified as cancer treatment, the *system* as the capacity of older adults with advanced cancer, and the system’s *responses* as symptoms. To characterize resilience in the context of symptom response to cancer treatment, four criteria were used: (1) the presence of none/mild symptom severity by the end of observation (positive outcome)^26^^,^^30^^,^^31^; (2) increased symptom severity compared to baseline (resist or not); (3) if there was increased symptom severity, whether there was symptom recovery; and (4) if recovered, what was the recovered severity compared to baseline (better than baseline, same as the baseline, or worse than baseline).

**Figure 1.**
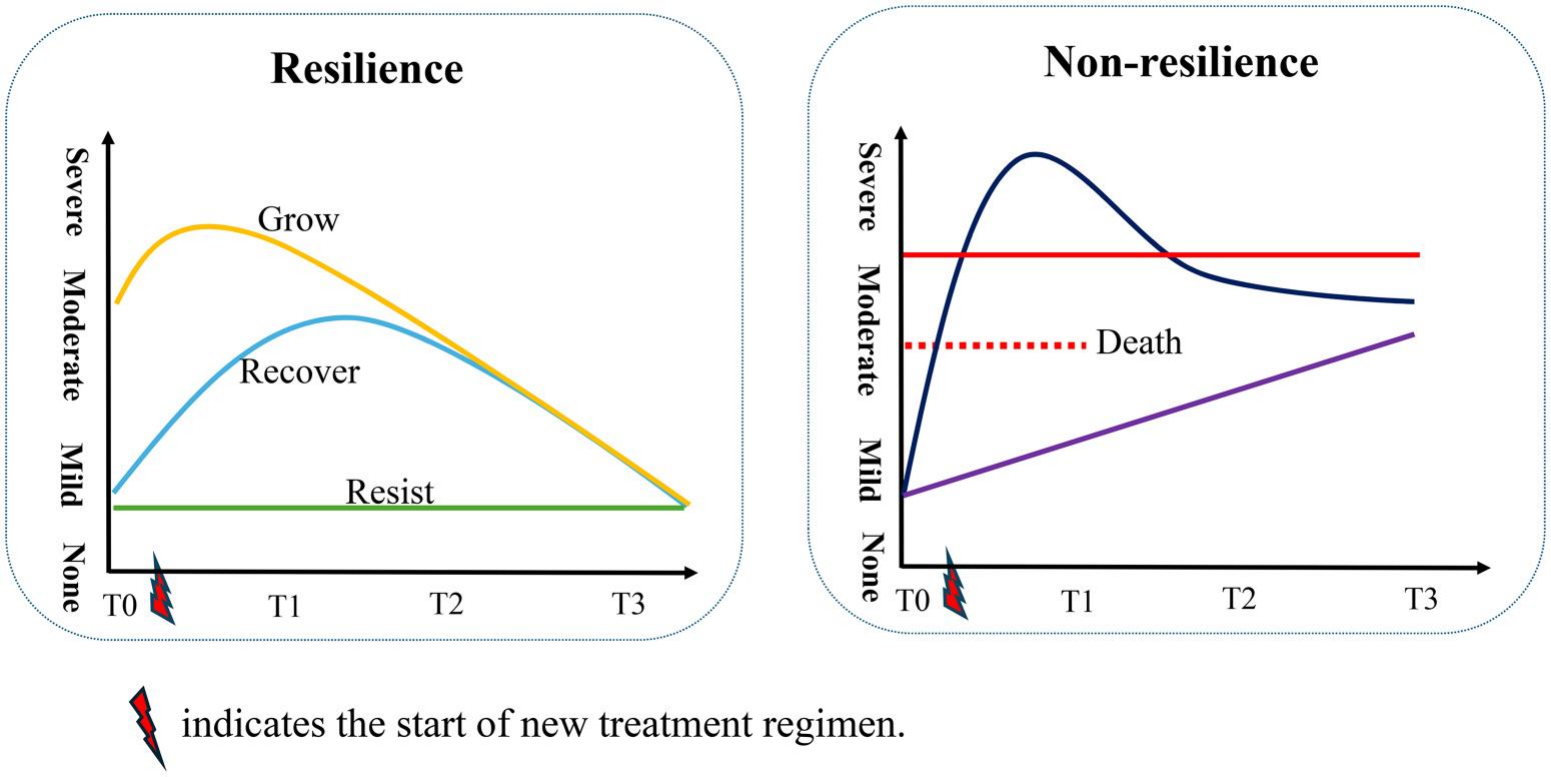
Example trajectories of resilience and non-resilience.

Based on the above four criteria, resilience in symptom response to cancer treatment can be identified by the presence of any one of three symptom trajectory patterns (Figure 1): (1) maintaining none/mild symptom severity (“resist”), (2) increasing from baseline none/mild to moderate/severe symptoms but returning to baseline none/mild severity (“recover”, same as baseline), or (3) starting with moderate to severe symptom severity but recovering to none/mild severity (“grow”, better than baseline). All other trajectories were characterized as non-resilience, such as maintaining consistently moderate or severe symptom severity over treatment, declining from baseline severe symptom to moderate severity, and increasing from mild to moderate severity (Figure 1). For example, patients whose symptom severity declined from severe at baseline to a moderate level over time demonstrated improvement but did not meet the above criterion 1, which defines a positive outcome as achieving none or mild symptom severity. As a result, they were classified as non-resilient. Additional rationale for the classification criteria is provided in Appendix S1. Additionally, patients who died during treatment completely lost the ability to resist, recover, or grow from stressors and were thus included in the non-resilience group. Figure 1 presents illustrative examples of potential symptom trajectories for conceptual clarity. The presence of any one of the three defined trajectory patterns is sufficient to indicate resilience. Not all three resilient trajectory patterns are expected to be observed within any single study, and the non-resilient trajectories shown are illustrative rather than exhaustive of all possible non-resilient patterns.

### Variables

#### Demographics and clinical characteristics

Demographic variables including age, gender, and race/ethnicity, education, income, and living status were collected via self-reported questionnaires. Clinical characteristics including cancer type and other treatment characteristics were extracted from medical records.

#### Resilience in symptom response to cancer treatment

Symptoms were assessed using PRO-CTCAE in the GAP 70+ study prior to the initiation of a new treatment regimen and at 4-6 weeks, 3 and 6 months later. The symptom response to cancer treatment was indicated by a summary severity score of 24 symptoms. The development of the summary severity score and details on the 24 symptoms have been reported.^32^ The total score ranges from 0-96, and a higher score indicates worse symptom severity. Based on established thresholds, mean scores of 6.33 (SD = 3.44), 16.57 (SD = 4.32), and 29.80 (SD = 7.80) indicate low, moderate, and high symptom severity, respectively.^32^ Accordingly, a mean score below 6.33 corresponds to none or mild symptom severity in this study.^32^ Resilience in summary severity score was determined based on the above-described criteria.

#### Functional status

Functional status was assessed with the instrumental activities of daily living (IADL) scale at the same four assessment points as PRO-CTCAE. IADL is a 7-item subscale of the daily living domain from the Older Americans Resources and Services (OARS) multidimensional functional assessment questionnaires.^33^^,^^34^ It assesses independence in daily living activities, including the ability to use a telephone, transport long distances, go shopping, prepare meals, do housework, take medicine, and handle money. The total score ranges from 0-14 points, and a higher score indicates a better functional status.^33^^,^^34^

### Statistical analysis

Patient characteristics were delineated with descriptive statistics. A two-step approach was used to classify resilience and non-resilience in symptom response to cancer treatment. First, patients who died within 6 months were considered to have non-resilience trajectories. Second, growth mixture models (GMM) were employed to identify symptom trajectories in the summary severity score of 24 symptoms for the remaining patients who were alive at 6 months.^35^ GMM allows us to group patients with similar patterns of symptom severity change over time, thereby identifying clinically meaningful subgroups with different symptom trajectories. In our preliminary analysis, after excluding patients who died, no significant differences were observed between patients with complete data and those with missing data at these time points, suggesting that the missing data were likely missing at random. Under this assumption, GMM can appropriately accommodate missing observations while estimating symptom trajectory patterns. The identified trajectories were then reviewed and labeled as resilience or non-resilience according to the definition. Trajectories in the GMM were estimated using maximum likelihood, testing models with up to seven trajectory groups.^36^ Both linear and quadratic terms were included in the model, with growth factor variances fixed to zero, to ensure precise estimation of trajectory shapes. Guided by the resilience framework, the best number of trajectory group was determined not only by the model fit indices, such as Akaike Information Criterion (AIC) and Bayesian Information Criterion (BIC) as detailed in Table 1^36^^,^^37^, but also on their consistency with predefined resilience criteria to ensure meaningful identification of resilience from the trajectory patterns. To further ensure the clinical relevance of identified trajectories alignment with the resilience framework, the trajectories with the best number of trajectory group were reviewed and discussed with research team members (M.F. and E.C.). The symptom trajectories were estimated with Mplus version 8.0.

**Table 1.** Considerations in selecting the number of classes for symptom severity trajectory.

Considerations	Interpretations
Model fit indices	
** BIC**	The lower, the better model fit
** AIC**	The lower, the better model fit
** LMR-LRT**	A significant P value indicates a k + 1-class model fits better than a k-class model
**Classification quality**	
** Entropy**	The closer to 1, the better distinction among classes
** Posterior possibility**	The closer to 1, the better distinction among classes
** Class size**	No less than 1% in each class, and greater than 5% in each class is preferred
**Parsimony**	A model with too many classes may not be as useful as a model with fewer classes
**Theoretical justification**	Identified trajectories fit with the resilience framework
**Interpretability**	Clinical meaningful, or useful in practice

Abbreviations: AIC, Akaike Information Criterion; BIC, Bayesian Information Criterion; LMR-LRT, Lo-Mendell-Rubin likelihood ratio test.

A longitudinal linear mixed model (LLMM) was employed to compare differences in functional status over 6 months among those with and without resilience in symptom response to cancer treatment. Restricted maximum likelihood estimation was used, assuming that the missing IADL data occurred at random. In the LLMM, the dependent variable was the total IADL score (range 0-14 points); group (resilience or non-resilience), time (prior to the initiation of a new treatment regimen, 4-6 weeks, 3 and 6 months after the treatment initiation), and the interaction between group and time were entered as fixed effects; patient’s ID was entered as a random effect. The analysis was controlled for the study arm and the cluster, since the data were from a cluster-randomized clinical trial. A sensitivity analysis was conducted by repeating the analysis specifically on only the sample of patients who remained alive at 6 months. The LLMM was conducted with the PROC MIXED procedure in SAS, and a two-sided significance level was set at 0.05.

## Results

### Sample characteristics

A total of 710 patients who provided at least one PRO-CTCAE data were included in the study. Table 2 presents the characteristics of the 710 included patients. The average age was 77.2 years (standard deviation [SD]=5.4, range 70-96). Among them, 43.4% were female and 62.8% were married or had a domestic partner. The most prevalent cancer types were gastrointestinal (*n* = 246; 34.6%) and lung cancer (*n* = 177; 24.9%). The majority of patients (88.0%) received at least one chemotherapy agent, with multiple-agent chemotherapy being the most common regimen type (46.2%).

**Table 2.** Characteristics of the 710 patients included.

Variables	Classifications	*N* (%)
**Study arm**	GA intervention	344 (48.5)
	Usual care	366 (51.5)
**Age (years)^a^**		77.2 (5.4)
**Sex**	Male	402 (56.6)
	Female	308 (43.4)
**Race/ethnicity**	Non-Hispanic White	623 (87.7)
	Other racial/ethnic group	86 (12.1)
	Missing	1 (0.1)
**Education**	≤High school	351 (49.4)
	>High school	359 (50.6)
**Marital status**	Married/domestic partner	446 (62.8)
	Other	264 (47.2)
**Income**	≤$50 000	366 (51.5)
	>$50 000	190 (26.8)
	Decline to answer	154 (21.7)
**Living status**	Live alone	171 (24.1)
	Live with others	539 (75.9)
**Number of GA impairments^a^**		4.5 (4.0)
**Prior chemotherapy**		185 (26.1)
**Cancer type**	GI cancer	246 (34.6)
	Lung cancer	177 (24.9)
	GU cancer	109 (15.4)
	Other diagnoses	178 (25.1)
**Treatment type**	Single-agent chemotherapy	146 (20.6)
	Multiple agent chemotherapy	328 (46.2)
	Chemotherapy and other agents	151 (21.3)
	Non-chemotherapy	85 (12.0)
**Standard treatment^b^**	Standard	430 (60.6)
	Non-standard	280 (39.4)

^a^Mean and standard deviation.

^b^Standard treatment refers to the therapeutic regimen that adheres to standard dose and scheduling outlined by NCCN guidelines or experimental therapeutics documented in published phase II/III trials.

Abbreviations: GA, geriatric assessment; GI, gastrointestinal; GU, genitourinary.

### Identification of resilience in symptom response to cancer treatment

#### Trajectories of summary severity score

Among the 710 patients included, 706 provided data on the summary severity score prior to initiating a new treatment, 621 at 4-6 weeks, 521 at 3 months, and 451 at 6 months. The mean summary severity score was 14.0 (SD = 9.3) at baseline, increased to 16.0 (SD = 9.6) at 4-6 weeks, and then slightly declined to 15.4 (SD = 9.4) at 3 months and 15.3 (SD = 9.1) at 6 months (Figure S1). Over the six-month follow-up period, 189 patients died, leaving 521 patients alive at 6 months.

The GMM analysis utilized data on the summary severity score from 521 patients who were alive at 6 months. The results of the model comparison from one to seven trajectory groups (ie, latent classes) are presented in Table 3. The AIC and BIC values decreased from the one-trajectory to the seven-trajectory model, indicating improved model fit with increasing numbers of trajectory groups. Trajectories consistent with the resilience framework were first observed in the four-group solution. Although models with five or more trajectory groups demonstrated further reductions in AIC and BIC, each included at least one group comprising approximately 1% of the sample, limiting interpretability and stability. Therefore, the four-trajectory model was selected as the optimal solution based on a balance of statistical fit, trajectory group size, and conceptual alignment with the resilience framework. For the four-group model, average posterior probabilities of group membership ranged from 0.800 to 0.934, indicating good to excellent classification quality. Within individual trajectory groups, the proportion of participants with lower classification certainty (posterior probability <0.70) ranged from 6.9% to 31.0% (19.4%, 6.9%, 31.0%, and 24.8% for Groups 1-4, respectively), reflecting variability in classification certainty across trajectory groups related to sample heterogeneity. Model entropy was 0.722, indicating good separation among the four trajectory groups.

**Table 3.** Model fit statistics for growth mixture models identifying one to seven symptom severity trajectory groups.

Group	AIC	BIC	*P*	Entropy	Posterior possibility	Trajectory group size (%)						
						Group 1	Group 2	Group 3	Group 4	Group 5	Group 6	Group 7
**1**	13970.7	14000.5	NA	NA	1.000	521 (100.0)						
**2**	13401.0	13447.8	.005	0.805	0.919-0.947	147 (28.2)	374 (71.8)					
**3**	13144.3	13208.2	<.001	0.843	0.912-0.956	192 (36.9)	30 (5.8)	299 (57.4)				
**4**	13099.8	13180.7	.170	0.722	0.800-0.934	144 (27.6)	29 (5.6)	126 (24.2)	222 (42.6)			
**5**	13057.7	13155.6	.383	0.768	0.800-0.941	222 (42.6)	5 (1.0)	113 (21.7)	25 (4.8)	156 (29.9)		
**6**	13026.9	13141.8	.091	0.789	0.808-0.922	12 (2.3)	218 (41.8)	116 (22.3)	5 (1.0)	27 (5.2)	143 (27.4)	
**7**	12995.9	13127.9	.109	0.810	0.824-0.998	15 (2.9)	25 (4.8)	5 (1.0)	138 (26.5)	117 (22.5)	216 (41.5)	5 (1.0)

Abbreviations: AIC, Akaike Information Criterion; BIC, Bayesian Information Criterion; *p* values were estimated from the Lo-Mendell-Rubin likelihood ratio test.

#### Resilience or non-resilience classification

**Figure 2.**
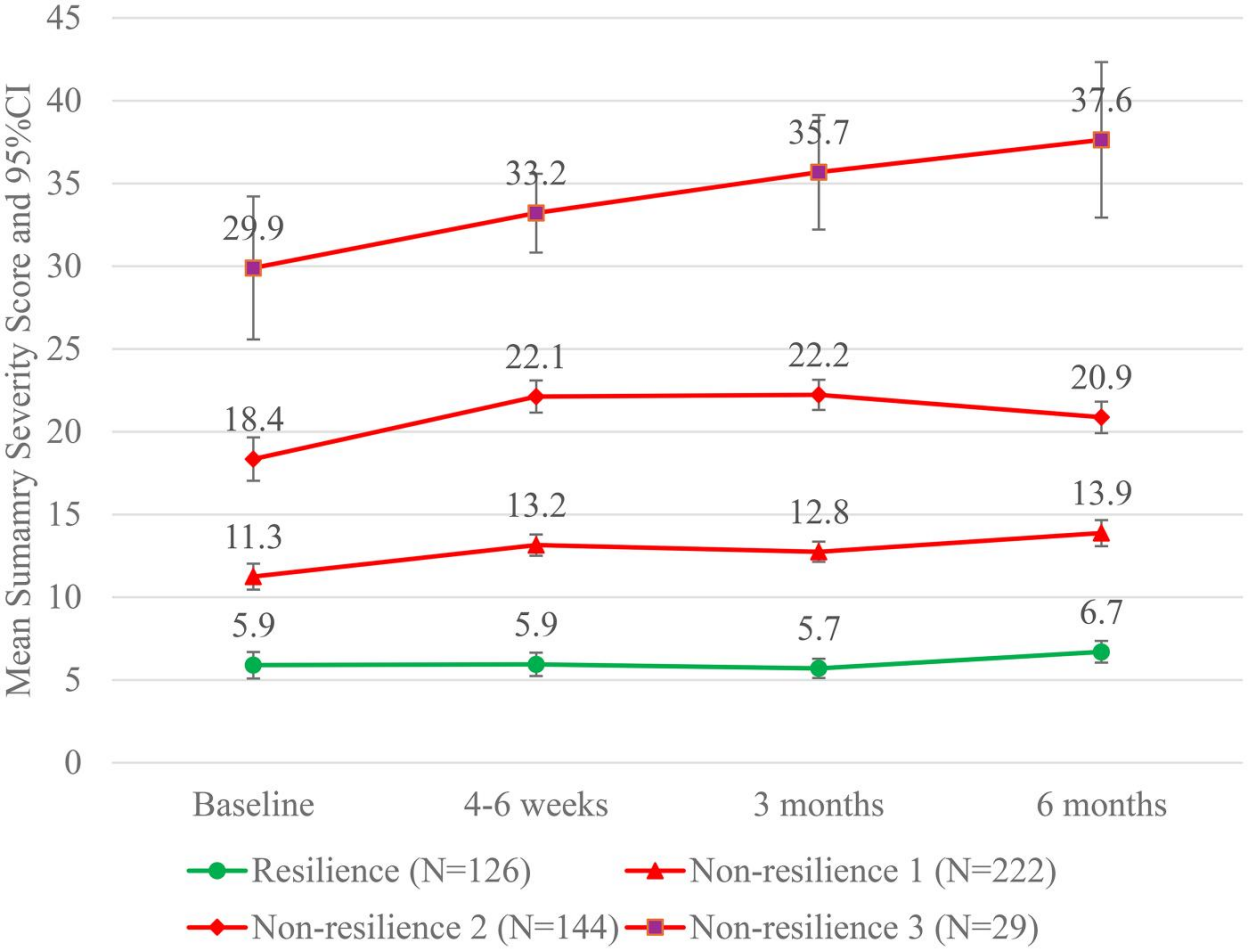
Resilience classification in the trajectories of summary symptom severity score. Data were collected at baseline (before initiating a new treatment) and at 4-6 weeks, 3 months, and 6 months later; trajectory modeling included participants alive at 6 months (*n* = 521), with observed data available for 517, 499, 468, and 445 participants at each respective time point.

Figure 2 illustrates the four trajectories identified using GMM. Only one (“resist”) trajectory (green line, *n* = 126) was identified, which consistently exhibited mild symptom severity, indicating resilience in symptom response to cancer treatment. No trajectories reflecting recovery (“recover”) or improvement (“grow”) were identified. In contrast, patients in the other three trajectories (red lines, total *n* = 395) reflected non-resilient symptom patterns. Among these, one small subgroup (*Non-resilience 3*, *n* = 29) demonstrated severe symptom burden at baseline with symptom severity steadily increasing over time. The other two trajectories (*Non-resilience 1*, *n* = 220; *Non-resilience 2*, *n* = 143) began with moderate symptom severity and showed slight fluctuations during follow-up without recovery to none or mild symptom severity or progression to severe symptom severity. Because symptom severity did not return to none or mild levels by 6 months (does not meet criterion 1), these three trajectories were classified into the non-resilience group. Additionally, 189 patients who died within 6 months were also classified into the non-resilience group. Thus, the resilience rate in symptom response to cancer treatment was 17.7% (126/710), while the non-resilience rate was 82.3% (584/710).

### Association of resilience in symptom response to cancer treatment and functional status

Among the 710 patients included in this study, IADL scores were available for 710 patients at baseline, 622 at 4-6 weeks, 522 at 3 months, and 452 at 6 months. The mean IADL score was 12.3 (SD = 2.5) at baseline, declined to 11.8 (SD = 2.8) at 4-6 weeks and 12.0 (SD = 2.6) at 3 months, and then increased to 12.2 (SD = 2.6) at 6 months (Figure S2). At 6 months, 32.5% of patients had IADL scores that were unchanged from baseline; this proportion was substantially higher in the resilient group (62.7%) than in the non-resilient group (26.0%). A longitudinal mixed-effects model revealed significant associations between IADL scores over 6 months and resilience group (*P* <.001), time (*P* <.001), and their interaction (*P* <.001), adjusting for study arm (fixed effect) and cluster (random effect).

**Figure 3.**
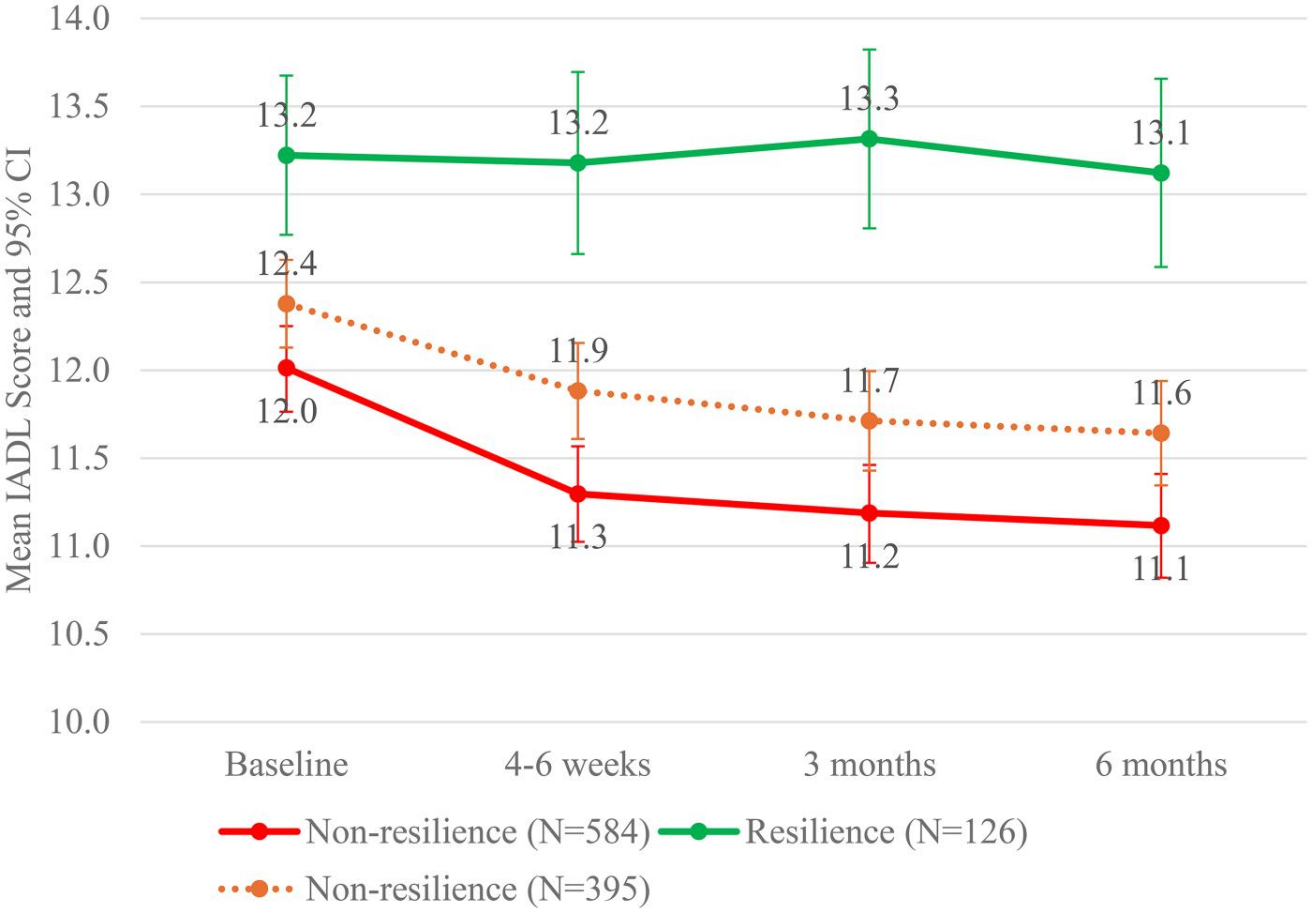
IADL scores of patients in the resilience and non-resilience groups over 6 months. Solid lines represent estimates based on the total sample (*N* = 710), while dotted lines reflect estimates based on participants who were still alive at 6 months (*n* = 512). Resilience is determined by the summary severity score. IADL scores were estimated as marginal means from the longitudinal mixed-effect linear model. Data were collected at baseline (prior to the initiation of a new treatment) and at 4-6 weeks, 3 months, and 6 months later, with IADL data available for 710, 622, 522, and 452 participants at each respective time point. CI, confidence interval; IADL, instrumental activities of daily living.

Resilience in symptom response was associated with higher and more stable functional status. As illustrated in Figure 3, no significant changes in IADL scores were observed over time (all *P* > .05) in this group. Additionally, IADL scores in the resilience group were consistently higher than the score in the non-resilience group across all time points: 1.2 points higher at baseline (95% CI, 0.7-1.7), 1.9 at 4-6 weeks (95% CI, 1.3-2.4), 2.1 at 3 months (95% CI, 1.6-2.7), and 2.0 at 6 months (95% CI, 1.4-2.6) (all *P* <.001). In contrast, functional status declined at 4-6 weeks without recovering at 6 months in the non-resilience group. Within this group, IADL scores declined significantly from baseline: by 0.7 points at 4-6 weeks (95% confidence interval [CI]: 0.5-0.9), 0.8 points at 3 months (95% CI, 0.6-1.0), and 0.9 points at 6 months (95% CI, 0.7-1.1) (all *P* <.001). The overall mean difference between the two groups was 1.8 points (95% CI, 1.3-2.3, *P* <.001) over the 6 months. These patterns were consistent in a sensitivity analysis in a subgroup of patients who were alive at 6 months (*n* = 521).

## Discussion

This study is the first to examine resilience in symptom response to cancer treatment using the NIH-RCM framework in older adults with advanced cancer. The findings demonstrate the applicability of NIH-RCM in guiding the examination of variable symptom trajectories during cancer treatment. Resilience in symptom response to cancer treatment characterizes an ability to resist, recover, or grow from cancer treatment. It was associated with a higher functional status without functional decline during cancer treatment.

Only one in five older adults with advanced cancer exhibited resilience in symptom response to cancer treatment, indicating poor treatment tolerability.^38^ Advanced cancer and aging-related impairments are two known risk factors for increased symptoms.^3^^,^^39–42^ In this cohort, these two factors were likely to influence an individual’s symptom response to cancer treatment, lowering the resilience rate. Additionally, the intensity of chemotherapy regimens and insufficient supportive care systems may further diminish the likelihood of exhibiting resilience in symptom response during cancer treatment. Aligned with treatment guidelines,^43^^,^^44^ these findings highlight the urgent need for palliative and supportive care, as well as the integration of symptom monitoring and management into standard oncology practice to optimize symptom control and improve overall quality of life for this population.^11^^,^^45–47^

Resilience in symptom response was associated with higher functional status over six months, resisting or recovering from functional decline during treatment. Functional status typically deteriorates during cancer treatment,^20^^,^^23^^,^^24^ which is consistent with the observations in the non-resilience group, where function declined sharply within 4-6 weeks with no recovery at six months. This sharp decline may have been driven by increased symptom severity following the initiation of cancer treatment. This observation supports the relationship proposed in Kumpfer’s resilience framework, which suggests that resilience is associated with positive outcomes^25^^,^^26^ and aligns with the broader literature that associates more severe symptoms with worse functional outcomes in this population.^48^^,^^49^ Prior research has used various indicators to assess resilience, with functional status commonly used in older adults.^50–55^ The observed correlation between resilience, indicated by symptom response, and maintaining functional status suggests that these two indicators may reflect overlapping aspects of the same underlying construct. This interrelationship supports the notion that resilience can manifest through multiple, interconnected dimensions, such as symptom response and functional outcomes, within the context of cancer treatment.

The relationship between resilience in symptom response and functional status remained consistent in both primary and sensitivity analyses (excluding patients who died within 6 months). This study focuses on older adults with advanced cancer, a vulnerable cohort, among whom 26% passed away within 6 months, leading to a high attrition rate. In theory, older adults who died experienced an irreversible collapse in their symptom response to cancer treatment, reflecting an “exhaustion” state of the system–an indicator of non-resilience. The consistent patterns in resilience and functional status across primary and sensitivity analyses support the validity of classifying patients who died into the non-resilience group. Despite that, this approach should be subject to further evaluation in subsequent studies.

### Strengths and limitations

The strengths of this study lie in its novel approach to examining variable symptom trajectories through a resilience framework and its adaptation of this framework to the cancer treatment context. The NIH-RCM,^19^ a broad framework guiding resilience research across disciplines, requires adaptation to specific contexts, including cancer treatment. In this study, we tailored the framework by defining the three key components of resilience and establishing criteria for classifying resilience in symptom response. This approach moves beyond traditional vulnerability-based paradigms such as frailty. Whereas frailty conceptualizes vulnerability as a static condition that exists independent of external stressors, resilience emphasizes dynamic responses to stressors over time.^56–58^ This distinction is critical in cancer care. Rather than assessing symptoms at a single time point, this resilience framework focuses on dynamic changes in symptoms throughout the course of treatment, enabling a nuanced understanding of individual variability in symptom response.

Another strength of this study is that it addresses two limitations in previous resilience research: the failure to assess responses before the stressor and the unrepresentativeness of resilience based on single indicators (eg, individual symptoms like depression).^52^^,^^59^ First, we included all patients who started a new treatment regimen (stressor) and longitudinally assessed their symptoms both before and after (ie, response) the start of the new cancer treatment regimen. This comparison allows for a more representative reflection of symptom response to the stressor of cancer treatment. Second, we used a summary severity score of 24 symptoms to capture the system’s response to cancer treatment. This composite measure provides a more comprehensive view of the system’s response to cancer treatment, capturing multidimensional aspects of symptom experience. While this summary score offers a broader and potentially more representative indicator of symptom response than only one symptom, its validity as an indicator of resilience warrants further investigation. Future studies should continue to explore and compare alternative, multidimensional indicators of resilience to strengthen measurement approaches in this field.

There are limitations associated with implementing the NIH-RCM in this study. First, cancer treatment was regarded as the sole stressor. This assumption serves as an idealized approach to streamline the exploration of resilience within complex settings. However, it is important to acknowledge the presence of other stressors that can also elicit symptom responses, such as the underlying cancer itself and concurrent comorbidities. For example, 67% of patients had at least one comorbidity before cancer treatment.^29^ Moreover, patients received different types of treatment, including single-agent or multi-agent chemotherapy, chemotherapy and other agents, and non-chemotherapy,^29^ with many experiencing adjustments to their treatment plans during the study period.^60^ For instance, 18% of patients experienced early discontinuation at 3 months,^61^ and 35% required dose modifications at cycle one.^60^ Therefore, considering the complexity of cancer treatment in the context of older adults with advanced cancer, the resilience rates in symptom responses to cancer treatment should be interpreted with caution. Second, none/mild symptom severity was selected as the criterion for positive outcomes. However, patients with mild symptoms may still experience a lower quality of life compared to those without symptoms.^62–65^ Third, data was collected at only four time points, and this wide time interval between symptom assessment may not fully capture the dynamic changes in symptom severity over the six-month period.

## Conclusions

The NIH-RCM is a useful framework for guiding the examination of resilience in symptom response to cancer treatment in geriatric oncology settings. It provides a new perspective for understanding variable symptom trajectories. Resilience in symptom response was associated with higher and more stable functional status, with no significant decline observed throughout the course of treatment. Future research should explore how resilience in symptom response can guide the understanding of treatment tolerability and support effective symptom management during cancer treatment. Specifically, identifying protective and risk factors associated with resilience, including the stressor related factor (eg, type and intensity of treatment) and system related factors (eg, aging related factors, previous treatment, and social support), may shed light on its underlying mechanisms and guide the design of targeted interventions to promote resilient symptom responses among older adults with advanced cancer.

## Author contributions

Zhihong Zhang (Conceptualization, Data curation, Formal analysis, Funding acquisition, Investigation, Methodology, Visualization, Writing—original draft, Writing—review & editing), Eva Culakova (Methodology, Supervision, Writing—review & editing), Kah Poh Loh (Conceptualization, Supervision, Writing—review & editing), Kathi L. Heffner (Conceptualization, Supervision, Writing—review & editing), Mostafa Mohamed (Data curation, Writing—review & editing), Rachael G. Tylock (Data curation, Project administration, Writing—review & editing), Megan Wells (Data curation, Project administration, Writing—review & editing), Fiona A. Stauffer (Writing—review & editing), Supriya Mohile (Conceptualization, Funding acquisition, Resources, Writing—review & editing), and Marie Flannery (Conceptualization, Methodology, Resources, Supervision, Writing—review & editing)

## Supplementary material

Supplementary material is available at *The Oncologist* online.

## Funding

This work is supported by the Epsilon Xi Chapter of Sigma Theta Tau Research Grant Award. This work is also supported by the National Cancer Institute NCORP grant (UG1 CA189961 PIs: Mustian, Morrow); R01CA177592, U01CA233167, K24 AG056589 to S.M.

## Conflicts of interest

None declared.

## Supplementary Material

oyag094_Supplementary_Data

## Data Availability

The data underlying this article will be shared on reasonable request to the corresponding author.
