# Supplementary material for: Resilience in symptom response to cancer treatment: a new lens for examining variable symptom trajectories in older adults with advanced cancer
Source: Oncologist. 2026 Mar 20;31(6):oyag094. doi: 10.1093/oncolo/oyag094 (PMC13157340; doi:10.1093/oncolo/oyag094)
Supplement: oyag094_Supplementary_Data [file oyag094_supplementary_data.docx]

**Appendix 1**

**Rationale for Specifying Resilience in Symptom Response to Cancer Treatment**

The NIH-RCM is a general framework that guides resilience research across disciplines; the components and the classification of resilience need to be adapted to specific research contexts. In this study, the three components of resilience were defined and established for resilience in symptom response. For example, the presence of positive responses in the face of stressors is often viewed as a prerequisite of resilience, distinguishing it from general adaptation.^26,30,31^ Accordingly, we defined the presence of symptoms with none or mild severity in response to cancer treatment at the end of observation as the first classification criterion for resilience. In addition, the NIH-RCM is a simplified and idealized framework where all individuals share a similar unstressed baseline before the stressor, and there exists a better status (“grow”) than baseline in target responses. However, in the context of symptom response to cancer treatment in older adults with advanced cancer, patients may already be experiencing symptom severity at baseline, making none or mild symptom severity as the best possible status. Therefore, we defined “grow” as a decrease in symptom severity from moderate or severe to none or mild. These additional specifications were necessary for accurate resilience classification in this context.

**Figure S1** Mean trajectories of summary symptom severity over six months in the total sample (N = 710).

Note: CI = Confidence Interval.

**Figure S2** Mean trajectories of IADL score over six months in the total sample (N = 710).

Note: IADL = Instrumental Activities of Daily Living; CI = Confidence Interval.
